# Allozyme comparison of two populations of *Rineloricaria* (Siluriformes, Loricariidae) from the Ivaí River, upper Paraná River basin, Brazil

**DOI:** 10.1590/S1415-47572009005000022

**Published:** 2009-01-30

**Authors:** Daniel M. Limeira, Erasmo Renesto, Cláudio H. Zawadzki

**Affiliations:** 1 Curso de Pós-graduação em Genética e Melhoramento, Universidade Estadual de Maringá, Maringá, PRBrazil; 2 Departamento de Biologia Celular e Genética, Universidade Estadual de Maringá, Maringá, PRBrazil; 3 Núcleo de Pesquisas em Limnologia Ictiologia e Aqüicultura, Departamento de Biologia, Universidade Estadual de Maringá, Maringá, PRBrazil

**Keywords:** allozymes, fish genetics, genetic distance, polymorphism, Loricariidae, Pisces

## Abstract

Two allopatric morphotypes of the genus *Rinelocaria* were compared through the allozyme electrophoresis technique: one morphotype, *R. pentamaculata*, from the Keller River in the middle stretch of the Ivaí River basin and the other, *R.* aff. *pentamaculata*, from the São João River in the upper portion of the Ivaí River basin. The morphotype from the São João River was collected upstream from the São João waterfall, which is about 80 m deep. Twelve enzymatic systems (AAT, ADH, EST, GCDH, G3PDH, GPI, IDH, LDH, MDH, ME, PGM and SOD) were analyzed, which allowed to score 22 *loci*. Only *lociAat-2, Est-3* and *Mdh-C* showed polymorphism. The two samples differed in allele frequencies at the three polymorphic *loci.* The average expected heterozygosity for all *loci* was 0.0806 ± 0.0447 in the Keller River sample. For the São João River morphotype, this value was 0.0489 ± 0.0350. Nei' s genetic identity and distance between the two populations were respectively 0.9789 and 0.0213. Wright's *F*_IS_ , *F*_IT_ and *F*_ST_ over all *loci* were estimated as 0.3121, 0.4021 and 0.1309, respectively. We consider that the two morphotypes represent species in *statunascendi.*

The Neotropical region harbors the most diversified ichthyofauna of the planet, corresponding to approximately 6,000 of the 13,000 species of freshwater fish. Of these, 4,475 have been described and there are probably 1,550 yet to be described (Reis *et al.*, 2003). The family Loricariidae of the order Siluriformes is the sixth largest family in the world (Nelson, 2006) and the second largest in Brazil. It encompasses 372 of the 2,300 species of valid freshwater fish (Buckup *et al.*, 2007).

The species concept has been a subject of debate for a long time and, according to Mayr (1977), species are groups of interbreeding natural populations that are reproductively isolated from other such groups with the same characteristics. The ambiguity and the difficulty of the species definition frequently lead to mistakes, especially in the case of sibling species. These are identical (or almost identical) species from a morphological point of view, but they are reproductively isolated (Mayr, 1977). When such species are analyzed by morphological characteristics only, the variation detected is not sufficient to consider them different species; however, genetic markers are capable of revealing reproductive isolation between them. On the other hand, geographically isolated populations sometimes acquire a degree of morphological divergence that would be usually found only in different species. Some of these populations, although morphologically very different, can interbreed in nature when geographic barriers disappear. Therefore, genetic markers have been used to supply evidence of reproductive isolation and the consequent decision as to whether morphologically different populations are different species.

Genetic difference is one of the ways to verify whether or not two populations belong to the same species. Nei (1972; 1978) developed statistics to calculate the identity and genetic distance between two populations. Thorpe and Solé-Cava (1994), after extensive revision of the literature, used values of genetic identity of Nei (1972) for the analysis of allopatric populations, including populations of the same species, populations of different species of the same genus, and species of different genera. According to these authors, the index of genetic identity of Nei (1972) varies between 0.85 and 1.00 between two populations of the same species and between 0.35 and 0.85 between two species of the same genus. Therefore, if the genetic identity between two taxonomic units is inferior to 0.85, it is improbable that they are conspecific. If it is inferior to 0.35, they should be considered species of a different genus.

Studies on isozyme electrophoresis have supplied important information about the genetic variability of natural populations, estimation of gene flow, elucidation of interspecific limits and the establishment of evolutionary relationships among different taxa (Solferini and Selivon, 2001). When the morphological criteria do not supply clear evidence for the validity of a genus, estimates of genetic distance can generate objective data (Thorpe, 1982; Nei, 1987).

*Rineloricaria pentamaculata* was described by Langeani and Araújo (1994), based on nine specimens collected from several locations in the upper Paraná River basin. Collections from the Ivaí River basin showed the presence of two morphologically different populations of *R. pentamaculata.* The main characteristic of *R. pentamaculata* is the presence of five dark dorsal stripes over the dorsum. It differs from congeners by having a naked snout tip, without platelets or odontodes, and an upper caudal-fin ray with an extension usually equal in length to the orbital diameter.

Considering that a waterfall can represent a geographical barrier to gene flow and that the two populations present morphological differences as regards the form of the snout, form of the lip, disposition of the dermal plates and form of the caudal fin, this work was done to verify whether or not these two populations belong to the same species.

Two samples of *Rineloricaria* from the Ivaí River basin (Figure 1) were collected between March and July 2006. Thirteen specimens (Figure 2) were collected in the Keller River, Marialva, Paraná State (23°38'30.59" S; 51°51'32.47" W) and 22 in the São João River, Prudentrópolis, Paraná State (25°4'29.95" S; 50°59'57.84" W). The São João River specimens were collected upstream from the São João waterfall (80 m high).

The Keller River sample was identified as *Rineloricaria pentamaculata* and the São João sample was identified as *R.* aff. *pentamaculata* because it shows morphological differences in relation to specimens from the Keller River*.* The main morphological differences between *R. pentamaculata* from the São João and Keller rivers are: I) a rounded *vs.* a pointed , more triangular snout tip; II) a lower lip distal border that is smooth or weakly serrate, *vs.* strongly serrate; III) each abdominal platelet being surrounded by skin, *vs.* abdominal platelets that are attached to each other and not surrounded by skin and; IV) an upper caudal-fin spine that usually does not extend beyond the branched ones, *vs.* an upper caudal-fin spine that is prolonged beyond the branched ones (the extension being similar in length to the eye diameter).

Muscle, liver, eye, stomach, heart, kidney and gill tissue samples were homogenized with a plastic stick in propylene tubes (1.5 mL) containing 100 μL of Tris-HCl buffer 0.02 M, pH 7.5 and centrifuged at 44,720 *g* at 4 °C for 30 min. Due to the presence of a great amount of fat in the liver, 100 μL of carbon tetrachloride (CCl_4_) was added to the tubes (Pasteur *et al.*, 1988).

Gels were prepared with 15% corn starch (Val *et al.*, 1981). Three buffer solutions were used: Tris 0.135 M/Citric acid 0.043 M pH 7.0 (Shaw and Prasad, 1970), Tris 0.18 M/Boric acid 0.1 M/EDTA 0.004 M pH 8.6 (Boyer *et al.*, 1963) and Tris 0.1 M/Maleic acid 0.1 M/EDTA 0.01 M pH 7.4 (Murphy *et al.*, 1996). The enzyme extract was applied to the gel using Whatman 3 MM® paper strips (4 mm x 8 mm) soaked with the samples. A voltage gradient of 40 V (measured at the extremities of the gel) was applied for 16 h under cooling. After electrophoresis, the gels were horizontally sliced lengthwise into two slabs, which were incubated using specific staining solutions according to Murphy *et al.* (1996).

The genetic variability was estimated according to the average heterozygosity (*He* and *Ho*) of Nei (1978). The homogeneity of the allele frequencies between populations was verified through a contingency chi-squared test. The unbiased genetic identity (I) and genetic distance (D) were calculated following Nei (1978). All of the estimates were calculated using the software POP GENE 1.31 (Yeh *et al.*, 1999). The significance of the differences between the heterozygosity values was evaluated by a t-test for paired data (Nei, 1987).

Voucher specimens were deposited in the ichthyological collection of the Núcleo de Pesquisas em Limnologia, Ictiologia e Aquicultura, Universidade Estadual de Maringá (NUP): *Rineloricaria pentamaculata*: NUP 2599, from the Keller River and *R.* aff. *pentamaculata*: NUP 4292, from the São João River. Twelve enzymatic systems (AAT, ADH, EST, GCDH, G3PDH, GPI, IDH, LDH, MDH, ME, PGM and SOD) were analyzed, which allowed to score 22 *loci*. Table 1 shows the analyzed enzymatic systems and the respective allele frequencies. In the sample from the Keller River, only *Aat-2*, *Est-3* and *Mdh-C* (13.64% of all *loci*) showed allele variation, with four, three and two alleles, respectively. In the sample from the São João River, only *Aat-2* and *Est-3* (9.09% of all *loci*) presented variation, with three alleles each.

Table 2 presents the values of observed and expected heterozygosities, and the effective number of alleles per *locus* for both samples. The average observed heterozygosity for all *loci* was 0.0475 ± 0.031 in the sample from the Keller River and 0.0411 ± 0.03 in the sample from the São João River, values that are not statistically different (t = 0.1787; 21 d. f.; p > 0.05). The average expected heterozygosity for the three polymorphic *loci* was estimated to be 0.0806 ± 0.0447 for the Keller River sample and 0.0489 ± 0.0350 for the São João River sample, values that are not statistically different (t = 1.095; 21 d. f.; p > 005). The sample from the Keller River was not in Hardy-Weinberg equilibrium for *Est-3* (χ^2^ = 14.0979; p = 0.003), while the *R. pentamaculata* sample from the São João River was in Hardy-Weinberg equilibrium for the three polymorphic *loci*. The two populations differed in the allele frequencies of *Est-3* (χ^2^ = 9.3778; p = 0.0092), *Aat-2* (χ^2^ = 15.56; p = 0.0014) and *Mdh-C* (χ^2^ = 29.46; p = 0).

Zawadzki *et al.* (2004) found all of the polymorphic *loci* of four populations of *Hypostomus* of the Keller River to be in Hardy-Weinberg equilibrium. Paiva *et al.* (2005) also detected Hardy-Weinberg equilibrium for three species of *Hypostomus* of the Maringá Stream. A great number of factors can deviate a population from Hardy-Weinberg equilibrium for a given *locus*, *e.g.* inbreeding, assortative mating, natural selection and gene flow (Nei, 1987), in addition to small sample size. Similar values of allele frequencies at the same *locus* turn the effective number of alleles (Ae) similar to the obtained number (Table 2). The value of Ae could have contributed to an increase in *He*, even though the number of polymorphic *loci* in our sample is small.

The value of *He* for other species of freshwater fish is reported as 0.051 (Ward *et al.*, 1992), when these were analyzed with the same 12 enzymatic systems as in our study. The average of *He* was 0.0806 in the sample from the Keller River, which is larger than the average for species of fish in general. However, in the population from the São João River, the value of *He* (0.0489) is smaller than the average estimated by Ward *et al.* (1992). The high heterozygosity estimated for the population of the Keller River results from the fact that this population has three polymorphic *loci*, while the population of the São João River presented only two. The high heterozygosity of Keller River population seems paradoxical, because that population was polymorphic at only 13.64% of the *loci*. Paiva *et al.* (2005), found 20% polymorphic *loci* for the population and *Hypostomus strigaticeps* of the Maringá Stream; however, the expected heterozygosity was only 0.028. A similar fact occurred with a *Hypostomus* sp. 3 population collected in the Itaipu Reservoir analyzed by Zawadzki *et al.* (2005), where these Loricariidae showed 24% of polymorphic *loci,* despite an expected heterozygosity was 0.048. The relatively high heterozygosity estimated in our work, when compared to the above studies, can be explained by the similarity among the allele frequencies in the analyzed polymorphic *loci*. The value of expected heterozygosity is maximal when the allele frequencies are similar and indeed, at *Mdh-C* we observed similar frequencies for the alleles *Mdh-C* (*a*) and *Mdh-C* (*b*) (0.4583 and 0.5417, respectively). In Paiva *et al.* (2005) and Zawadzki *et al.* (2005), the allele frequencies at the polymorphic *loci* were quite divergent, which explains the lower *He* value.

The *He* values estimated for species of Loricariidae have high variation, as found in several *Hypostomus* populations. The currently available results show that the family Loricariidae contains species with larger genetic variability than others, *i.e.* 0.011 and 0.017 for two populations of the Iguaçu River basin (Zawadzki *et al.*, 1999), 0.000 and 0.107 for two species of the Paraná River basin (Paiva *et al.*, 2005 and Zawadzki *et al.*, 2005, respectively), and 0.000 and 0.172 for *H. albopunctatus* and *H.hermani*, respectively (Paiva S, MSc Dissertation, Universidade Estadual de Maringá, 2006).

Wright's *F*_IS_, *F*_IT_ and *F*_ST_ per polymorphic *locus* were statistically different from zero, which indicates a significant excess of homozygotes. The averages over all *loci* were estimated as *F*_IS_ = 0.3121, *F*_IT_ = 0.4021 and *F*_ST_ = 0.1309, respectively. Nei's genetic identity and genetic distance were estimated as 0.9789 and 0.0213, respectively.

The *F*_IS_ value represents the level of gene fixation within subpopulations. *F*_IT_ represents the level of gene fixation present in the total population (ignoring the subdivision). The *F*_ST_ value indicates the genetic differentiation between two populations (Wright, 1978). Except for *Mdh-C* in the population of the Keller River, *F*_IS_ and *F*_IT_ values indicate a significant excess of homozygotes, while *F*_ST_ values show that 13.09% of the total heterozygosity is due to the differentiation between the two *Rineloricaria* populations analyzed. The excess of homozygotes is possibly a result of inbreeding, since *R. pentamaculata* is a sedentary species.

Values of *F*_ST_ above 0.25 can be considered indicative of a great genetic differentiation, whereas values between 0.15 and 0.05 indicate a moderate and below 0.05 a small differentiation between populations (Wright, 1978). Following Wright's criterion, *R. pentamaculata* of the Keller River and *R*. aff *pentamaculata* of the São João River show a moderate genetic differentiation. The values of *F*_ST_ are statistically different from zero for the three polymorphic *loci* (χ^2^ = 15.52, χ^2^ = 8.43, χ^2^ = 25.26; for *Aat-2*; *Est-3* and *Mdh-C*, respectively). These values show that the two populations are genetically different at these three *loci*; however, the average for the 22 *loci* turns the differentiation between the two populations moderate.

According to Thorpe and Solé-Cava (1994), significant variation at any *locus* represents a barrier to the gene flow for sympatric populations and may result in, at least partial reproductive isolation. In organisms with sexual reproduction, this variation indicates that two populations should be considered different species.

*Rineloricaria pentamaculata* of the Keller River and *R*. aff *pentamaculata* of the São João River of the Ivaí River basin are isolated geographically. As they differ morphologically, as well as in allele frequencies at the polymorphic *loci*, it is probable that they represent species in *statu nascendi* (Dobzhansky, 1970), *i.e.* in the process of genetic divergence to become two distinct species.

**Figure 1:**
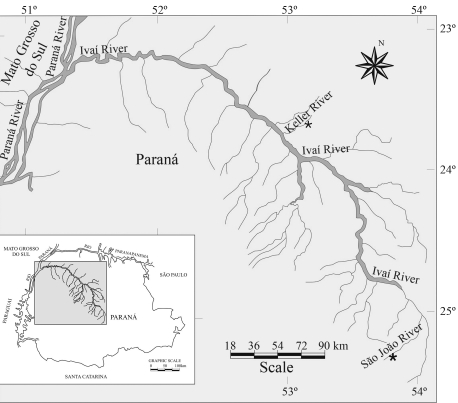
Ivaí River basin showing the collection sites.

**Figure 2:**
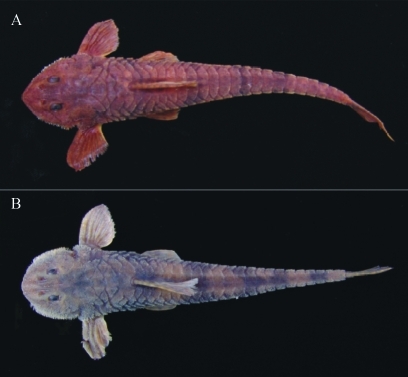
*Rineloricaria pentamaculata* of the Keller River, Ivaí River basin (A). *Rineloricaria* aff. *pentamaculata* of the São João River, Ivaí River basin (B).

## Figures and Tables

**Table 1:** Allele frequencies at 22 *loci* of *Rineloricaria pentamaculata* from Keller River and *Rineloricaria* aff. *pentamaculata* from São João River, Ivaí River basin.

Locus	Allele	Keller (n = 13)	São João (n = 22)	Tissue	*Buffer*
*Aat-1*	*a*	1*.*0000	1*.*0000	L	*TEM*
*Aat-2*	*a*	0*.*4615	0*.*7619	L	*TEM*
	*b*	0*.*0769	0*.*1667		
	*c*	0*.*2692	0*.*0714		
	*d*	0*.*1923	-		
*Acp-1*	*a*	1*.*0000	1*.*0000	L	*TC*
*Acp-2*	*a*	1*.*0000	1*.*0000	L	*TC*
*Adh*	*a*	1*.*0000	1*.*0000	L	*TBE*
*Est-1*	*a*	1*.*0000	1*.*0000	L	*TBE*
*Est-2*	*a*	1*.*0000	1*.*0000	L	*TBE*
*Est-3*	*a*	0*.*4615	0*.*3571	L	*TBE*
	*b*	0*.*0385	0*.*3571		
	*c*	0*.*5000	0*.*2857		
*Gdh*	*a*	1*.*0000	1*.*0000	L	*TEM*
*G3pdh-1*	*a*	1*.*0000	1*.*0000	M	*TC*
*G3pdh-2*	*a*	1*.*0000	1*.*0000	M	*TC*
*Idh*	*a*	1*.*0000	1*.*0000	M	*TC*
*Ldh-A*	*a*	1*.*0000	1*.*0000	M	*TC*
*Ldh-B*	*a*	1*.*0000	1*.*0000	M	*TC*
*Mdh-A*	*a*	1*.*0000	1*.*0000	M	*TC*
*Mdh-B*	*a*	1*.*0000	1*.*0000	M	*TC*
*Mdh-C*	*a*	0*.*4583	1*.*0000	M	*TC*
	*b*	0*.*5417	-		
*Me-1*	*a*	1*.*0000	1*.*0000	M	*TC*
*Me-2*	*a*	1*.*0000	1*.*0000	M	*TC*
*Pgm*	*a*	1*.*0000	1*.*0000	M	*TC*
*Gpi-1*	*a*	1*.*0000	1*.*0000	M	*TC*
*Gpi-2*	*a*	1*.*0000	1*.*0000	M	*TC*

L = liver; M = muscle; n = number of specimes; TEM = Tris/EDTA/ Maleate; TC = Tris/Citrate; TBE = Tris/Borate/EDTA.

**Table 2:** Observed (*Ho*) and expected heterozygosity (*He*) per polymorphic *locus* and the average over 22 *loci*, and effective number of alleles (Ae) for *Rineloricaria pentamaculata* of the Keller River and *Rineloricaria* aff. *pentamaculata* of the São João River, Ivaí River basin.

Locus	Keller (n = 13)				São João (n = 22)		
	*Ho*	*He*	*Ae*		*Ho*	*He*	*Ae*
*Aat-2*	0.3846	0*.*6985	3.0450		0*.*3333	0*.*3961	1.6303
*Est-3*	0*.*0769	0*.*5569	2.1529		0*.*5714	0*.*6794	2.9697
*Mdh-C*	0*.*5833	0*.*5181	1.9862		0*.*0000	0*.*0000	1.0000
Average	0*.*0475	0*.*0806	1.1902		0*.*0411	0*.*0489	1.1182
SE	0*.*031	0*.*0447	0.2538		0*.*030	0*.*0350	0.2384

SE = Standard error.
